# Supplementary material for: Hyperspectral imaging of microwave metasurfaces with deeply subwavelength resolution
Source: Nat Commun. 2025 May 17;16:4612. doi: 10.1038/s41467-025-59814-y (PMC12085632; doi:10.1038/s41467-025-59814-y)
Supplement: Supplementary file 1 — Supplementary Information [file 41467_2025_59814_MOESM1_ESM.pdf]

# Hyperspectral imaging of microwave metasurfaces with deeply subwavelength resolution: supplemental document

This supplementary document provides supporting materials for the article 'Hyperspectral imaging of microwave metasurfaces with deeply subwavelength resolution'. It includes an expanded description of the imaging method, experimental setup and modelling of the layered metasurface, in addition to discussion of the imaging system resolution and extra experimental data to support the main text.

## 1. SINGLE-PIXEL IMAGING

The following discussion includes some additional clarifications beyond those discussed in the main text. In single-pixel imaging (a field intimately related to computational ghost imaging), the lack of spatial resolution in the single 'bucket' detector is overcome by applying time-varying spatial modulations to the unknown light field [1, 2]. By combining the known sequence of patterns masking the field or object, with the associated transmitted (or reflected or scattered) light, measured with a single element detector, an image may be formed. Mathematically the process may be described as follows by reducing the dimensions of the object reconstruction **o** from $N \times N$ pixels to a vector of $N^2 \times 1$ pixels:

$$\mathbf{o} = \mathbf{P}^{-1}\mathbf{s}. \tag{S1}$$

**P** is the illumination basis, a matrix with columns containing the vectorised 2D projections and **s** is the vector of bucket signals, measuring the spatial overlap between the projections (which need not always match the basis **P** [3]) and the object or field.

In the case that the illumination basis is orthogonal, such as for the raster or popular Hadamard bases, the inverse of **P** is simply its transpose. In the intuitive case where **P** is the raster basis, i.e. a single 'on' pixel moving with each pattern, the bucket signal vector **s** directly becomes the image when reshaped to $N \times N$. Due to the limited signal arising from one pixel (smaller than one antenna in this work), we opt for the more efficient Hadamard basis [4, 5], in which 50% of the pixels are 'on' for each pattern in the basis.

The number of projections needed to form a complete (i.e. not compressed) measurement of the object scales with the resolution via the total image pixel count $N^2$. As the orthogonal Hadamard patterns contain both +1 and -1 values, a differential measurement scheme between two all positive binary masks is employed [2]. This increases the total number of projections necessary by a factor of 2. For all images in this work the resolution is $128 \times 128$ pixels, therefore the number of projections per single-frequency image is 32,768.

## 2. EXPERIMENTAL DETAILS

A schematic of the imaging system is shown in Fig. 2(a) of the main text and labelled photographs of the system are shown in Fig. S1. A pair of waveguide-coupled lens horn antennas (18810-MB-12223 UBR140, Flann Microwave) are positioned to measure the TM microwave transmission or reflection at an angle of 35° to the normal of the photoactive metasurface shown in the inset of Fig. 1 of the main text. This illumination angle is convenient for allowing normal incidence photomodulation but is not essential. Any angle or polarisation configuration is possible subject to sufficient signal. For example, even normal incidence reflection measurements can be facilitated by the use of a directional coupler and an indium tin oxide beam combiner for the microwave and visible beams.

The microwave source is a frequency tuneable continuous wave signal generator (Agilent E8257C) set to 21 dBm output power, and the transmitted power is detected by a coaxial Schottky-diode (HP 8473B) and transmitted to a PC via DAQ card (NI PCIe-6341). The photomodulation is provided by a projection system utilizing a digital micromirror device (DLP7000) and a white

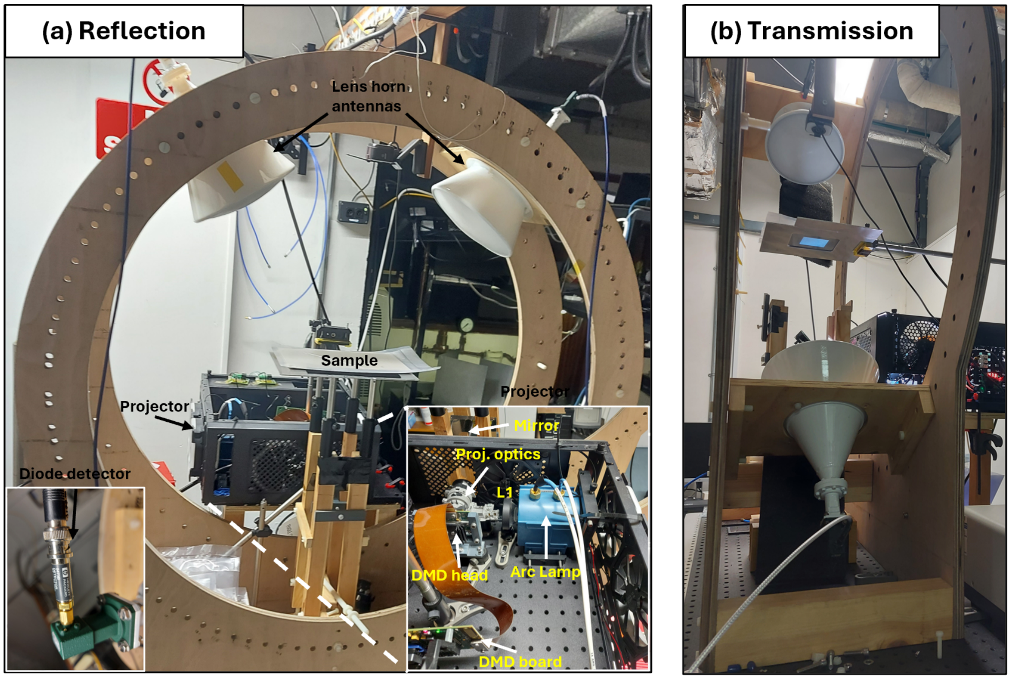


Fig. S1. Annotated photographs of the experimental apparatus in reflection mode (a) (used for Fig. S12) and transmission mode (b) (used for all other images). In (a) the blue patch cords lead to a VNA, for imaging one is connected to the signal generator output and the other to the diode detector shown inset.

xenon arc lamp (PE300BFM Excelitas), yielding a photoexcitation intensity of $\sim 100\ \mathrm{W/m^2}$ over an oversized area of the sample plane. The projection optics are taken from an optima H184X and coupling between the arc lamp and input light (homogenisation) pipe is performed via Aspheric Condenser Lens (L1), with f=32 mm from Thorlabs. The field of view (FOV) in the sample plane is varied between $4\times$ 4 mm and $80\times$ 80 mm as indicated, by changing the subsection of the DMD mirrors addressed and / or reconfiguring the projection optics to decrease the FOV where indicated in the text. When the FOV is reduced by replacing the default lens assembly with a lower magnification configuration, using an achromatic doublet with f = 75 mm (thorlabs), the illumination intensity is increased to $\sim 1000\ \mathrm{W/m^2}$.

To enable imaging, the projection system illuminates the silicon substrate (from the bottom of the inset of Fig. 1 of the main text) with a sequence of pre-determined optical Hadamard patterns. Through the photogeneration of free charge carriers, these patterns locally increase the conductivity of the wafer, increasing the absorptive damping for the adjacent resonators [6]. Using the differential measurement scheme discussed in Section 1, we determine the amplitude of the change in transmitted power for each pixel in the image, $|\delta T|$ following equation S1 (taking the modulus minimises some artefacts, such as negative values in the lower left corners of Fig. S6).

For the $128 \times 128$ pixel images (the default resolution) presented throughout this work, the number of projections per single-frequency image is 32,768, taking 1.6 s to acquire. We average 20 images (unless indicated otherwise) to maximise signal-to-noise ratio and assemble hyperspectral data cubes of dimensions $128 \times 128 \times \Delta F/f$ points, where $\Delta F$ is the frequency range plotted (typically between $11 - 20$ GHz) and $f$ the sampling interval of 100 MHz.

In photomodulation mediated RF and THz single-pixel imaging systems it is often beneficial to normalise the final images with respect to an image of the beams without any sample. This is done by recording images with only the silicon photomodulator present, and removes non-uniformity caused by the silicon, the visible excitation beam or the RF beam. The same modulator should be used and positioned in the exact same position for best results. The majority of our measurements use unpassivated 'off-the-shelf' silicon wafers with low carrier lifetimes, which receive the necessary boost in photomodulation efficiency due to their positioning in the near field of the metasurfaces. This means a normalisation measurement without the metasurface

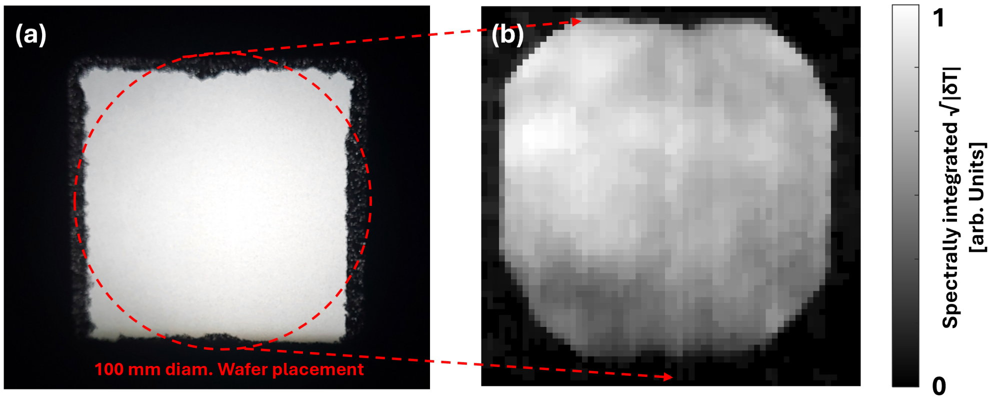


Fig. S2. (a) Photograph of the visible photoexcitation beam, taken with a smartphone camera and facilitated by a thin sheet of tracing paper in the sample plane. The metallic aperture has been lined with thin radiation-absorbent material. (b) Frequency averaged image of the square root of the transmission amplitude modulation over the range 12-18 GHz, using the 100 μm thick passivated wafer without any metasurface (TM 35°). This image is also sensitive to any spatial non-uniformity in the wafer carrier lifetime. A 3×3 median filter has been applied to this 64×64 resolution image to improve signal-to-noise ratio.

would exhibit very poor signal-noise-ratio. For this reason we present the unnormalised images after first ensuring adequate uniformity is present across the imaging FOV.

In Fig. S2(a) we show that the visible excitation beam is homogeneous, as it has passed through a light integration pipe within the projection optical assembly. In Fig. S2(b) we are able to confirm the broad coverage of the microwave beam by using the double-sided passivated 100 μm wafer used to produce Fig. S12, as it exhibits a higher intrinsic modulation efficiency. Some non-uniformity in signal is observed, which may be inherited from the passivated wafer. However it is clear that the microwave beam uniformity is sufficient for clearly identifying the localised defects discussed in this work.

For the experimental global transmission spectra shown in the main text (Fig. 1(a), Fig. 3 and Fig. 5), the source and detector are replaced with a two port VNA (Shockline MS46122B, Anritsu), the standard approach in the microwave regime. As the PCB section of the layered metasurface (100 x 100 mm) is smaller than the 150 mm diameter silicon wafer, a (100 x 100 mm) metallic aperture was used in all measurements to mask sections of the wafer without antennas. Note that this is only necessary for the VNA measurements, in order to avoid reduced spectral contrast from the bare silicon regions.

## 3. COMSOL MULTIPHYSICS MODELS

The simulation results presented in the main text and the supplementary document were obtained using COMSOL Multiphysics v6.1, with the exception of Fig. S10 in this supplementary document. The RF response of the metasurface was determined using a periodic model of the unit cell shown in Fig. 1 of the main text using the RF module with periodic boundary conditions on the sides of the modelling domain and periodic ports on the top and bottom of the modelling domain. The unit cell geometry is further specified in Fig. S3 and Tables S1 and S2.

The copper of the PCB was described using an Impedance Boundary Condition described by a fixed conductivity. It is known that the conductivity of the copper layer on PCBs is not the same as the bulk conductivity of copper due to surface roughness [7], with the conductivity depending upon both the degree of roughness and the frequency of the currents on the surface (and thus the frequency of any radiation interacting with the surface). From the data sheet for the I-tera MT 40 PCB the roughness is given as $Rz \leq 2.5$ μm, which at the central frequency used in this work corresponds to a conductivity of approximately $2.5\times10^{7}$ S/m. The permittivity of the PCB substrate as taken from the data sheet was $\varepsilon = 3.45 + 0.011i$, and its thickness was 0.51 mm.

Initial comparisons were undertaken between the modelled data and experimental transmission

Table S1. **Model lateral dimensions**

| Parameter | Symbol | Value [mm] |
|---|---|---|
| Unit cell width | $W_{cell}$ | 3.4 |
| Square 1 side length | $L_{sq1}$ | 1.349 |
| Square 2 side length | $L_{sq2}$ | 1.184 |
| Spiral 1 gap | $g_1$ | 0.2 |
| Spiral 2 gap | $g_2$ | 0.2 |
| Track width | $W_{track}$ | 0.16 |

Table S2. **Model layer properties**

| Layer | Thickness [µm] | Electromagnetic property | Value |
|---|---|---|---|
| Vac. bag wall | 100 | Relative permittivity | 3 + 0.009i |
| PCB substrate | 510 | Relative permittivity | 3.45 + 0.011i |
| Copper | 18 | Conductivity | 2.5 S/m |
| Silicon | 675 | Relative permittivity | 11.7 + 0.003i |

data for the spiral frequency selective surface *without* the addition of the Si wafer. In order to match the resonance frequency of the two modes the length of the spirals had to be increased relative to the original design, by 9 and 4 µm for the lower and higher frequency resonances respectively. This is likely due to a small degree of over-etching in the fabrication process.

For the models where the Si was included, the permittivity of the Si used was 11.7+0.003i (except where photoexcitation was included, in which case a Drude model was used as described below). The vacuum bag was also included in the model, with a thickness of 100 µm and permittivity of $\varepsilon = 3 + 0.009i$.

The charge carrier distributions shown in Fig. S4 for structured illumination and for uniform illumination as an input for producing Fig. S8 were obtained using COMSOL's semiconductor module. Since we use high resistivity, long charge carrier lifetime wafers in our experiments we describe the bulk lifetime as very long (100 ms) using COMSOL's built in Shockley-Reed-Hall bulk recombination model, but this value is rather arbitrary since surface recombination dominates. To model the charge carrier recombination at the surfaces of our Si modelling domain we incorporate

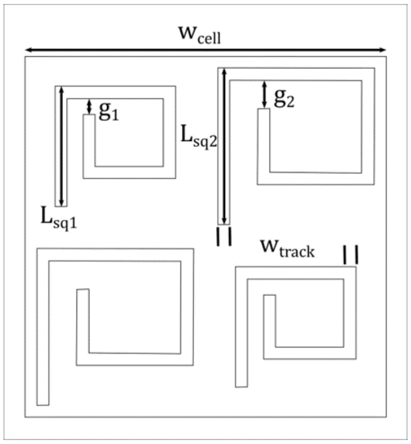


Fig. S3. Lateral dimensions of the modeled unit cell, with values provided in Table S1.

COMSOL's built-in surface recombination boundary condition, and subsequently altered the Surface Recombination Velocity in order to match the modelled lifetime of the charge carriers to those obtained from experimental measurements.

The periodic photoexcitation was modelled as a generation rate given by,

$$G = \frac{T k_a P_0 \lambda}{hc} \exp\left(-k_a x\right) \tag{S2}$$

where $T = 0.651$ is the proportion of incident light transmitted into the wafer (as calculated using Fresnel's equations), $k_a = 4\pi n_i/\lambda$ is the absorption coefficient with $n_i = 0.017$ being the imaginary part of the refractive index at a wavelength $\lambda$ of 625 nm, $P_0$ is the intensity of the photoexciting light "incident" on the surface, $h$ and $c$ are Plank's constant and the speed of light in vacuum respectively, and $x$ is the distance into the silicon.

The steady-state spatial charge carrier density distribution was subsequently solved for, and used with the following Drude model for the electrons and holes to obtain a spatially varying permittivity profile.

$$\varepsilon_{\mathrm{si}}(\omega) = \varepsilon_{\mathrm{bg}} - \frac{\omega_{pe}^2}{\omega(\omega + i\gamma_e)} - \frac{\omega_{ph}^2}{\omega(\omega + i\gamma_h)} \tag{S3}$$

where $\omega$ is the angular frequency of the radiation, $\varepsilon_{\mathrm{bg}} = 11.7 + 0.003i$ is the background permittivity of the silicon, $\omega_{p(e,h)} = \sqrt{(\Delta n)\, e^2 / \left(\varepsilon_0 m_{(e,h)}\right)}$ are the plasma frequencies for the electrons and holes with $m_e = 0.26 m_0$ and $m_h = 0.38 m_0$ being the conductivity effective masses. $\gamma_{(e,h)} = e/\left(m_{(e,h)}\mu_{(e,h)}\right)$ are the scattering rates of the electrons and holes, with $\mu_e = 0.145$ $\mathrm{m^2V^{-1}s^{-1}}$ and $\mu_h = 0.045$ $\mathrm{m^2V^{-1}s^{-1}}$ being the electron and hole mobilities, and $e$ and $\varepsilon_0$ are the electron charge and permittivity of free space respectively.

The COMSOL model files used throughout are available as part of the data set accompanying this work, from https://doi.org/10.5281/zenodo.15208969.

## 4. RESOLUTION

Whilst it is clear from the experimental images presented in the main text that the achieved resolution is easily sufficient to separate individual meta-atoms (sub-mm), accurately calculating the expected system resolution is no trivial matter. There are several relevant length scales here, the first being the size of the projected pixels themselves. For the largest FOV covered in the main text ($\sim 80 \times 80$ mm), in Fig. 4(a), these are $\sim$ 640 μm wide, well below the 1.5 cm to 3.0 cm wavelengths here. In addition, the lifetime of the photoexcited charge carriers in the wafer (30-50 μs) will determine a characteristic length scale over which they will diffuse before recombining, which is $\sim$ 200-300 μm for those wafers used in Fig. 2-5 of the main text [6]. Whilst this distance is representative, the contrast between photoexcited and non-photoexcited regions also depends on the thickness of the wafer through which the charge carriers are able to diffuse, shown in Fig. S4(a), and the field distribution of the resonance within the silicon, shown in Fig. S5. Based on the contrast in the imaginary part of the permittivity of the silicon observed in Fig. S4(a) (the change in the real part is minimal [6]) and the considerable penetration of the resonant fields towards the photoexcitation surface, we would expect an achievable resolution of $\sim$ 600 μm or below for this realisation of the imaging system. When the pixel size is reduced to 200 μm for Fig. 4(b) of the main text, the calculated permittivity distribution shows significantly reduced contrast, as shown in Fig. S4(b). Here it is clear that the achieved resolution will be worse than that suggested by the projected pixel size. However, there remains an additional subtlety.

The results of Fig. S4 are based on the steady-state solution under continuous photoexcitation, yet the experimental measurements are taken with projections held for just 50 μs. The result is that we expect the experimental permittivity distributions will have better contrast than indicated in Fig. S4. This is confirmed in the experimental data presented in Fig. S6, where images are compared for increasing measurement times, approaching the steady-state solution for several hundred microseconds. One can clearly see the apparent lateral size of the meta-atoms increase with integration time, due to increased charge carrier diffusion. This further supports the expectation that the achieved resolution is below $\sim$ 600 μm ($< \bar{\lambda}/30$).

In Fig. 6 of the main text a much thinner 50 μm thick 80 mm diameter silicon membrane (purchased from Silson Ltd) is used as the photoactive layer, surrounded by an annulus of 525 μm

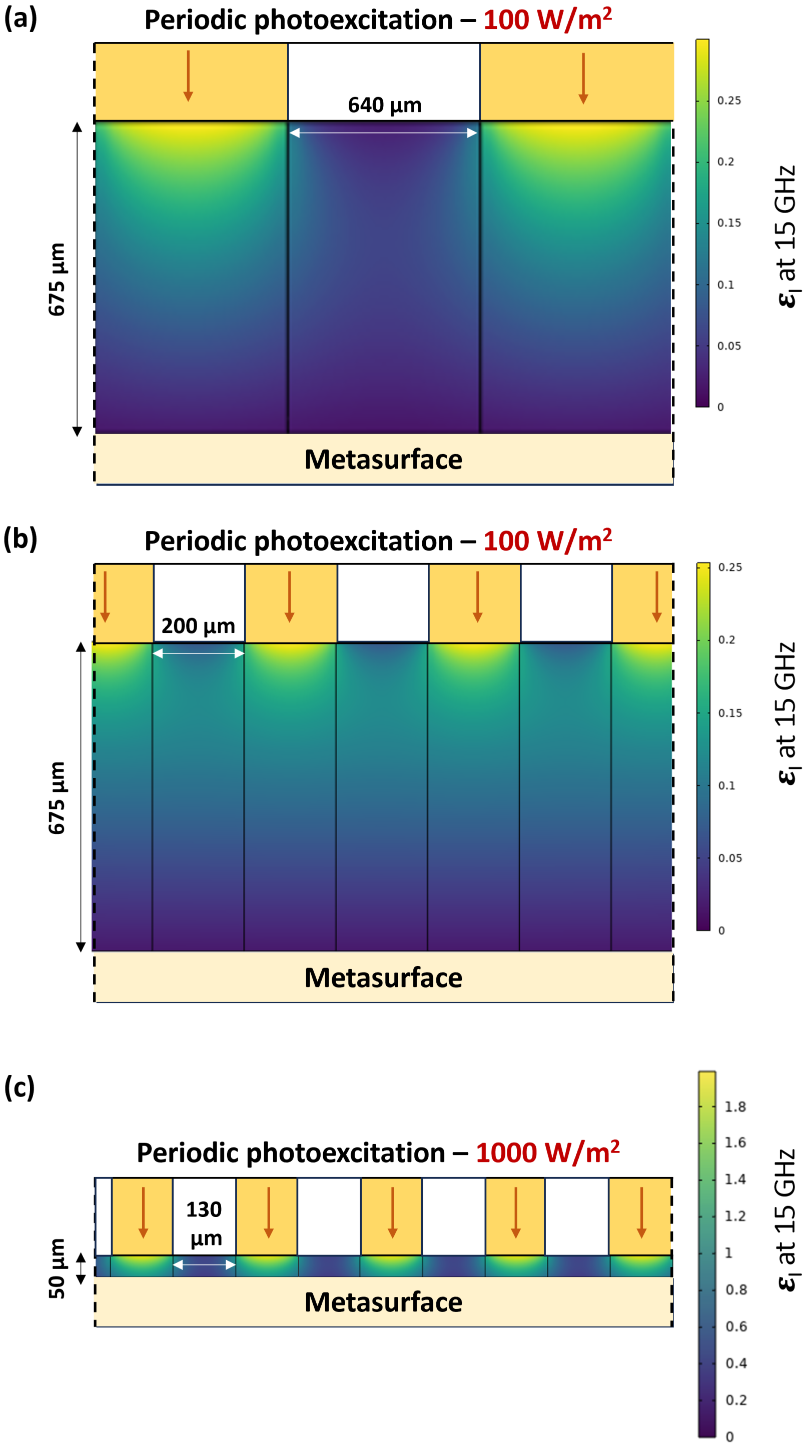


Fig. S4. Distribution of the imaginary part of the permittivity ($\epsilon_{|}$) of the silicon wafer upon photoexcitation, at 15 GHz. Results of COMSOL simulation described in Section 2. (a) For a photoexcitation of a 675 µm thick wafer with a lifetime of 30 µs with 1280 µm period, representing the resolution in Fig. 4(a) of the main text. (b) For a period of 400 µm, corresponding to double the pixel size in Fig. 4(b) of the main text. (c) For a 50 µm thick silicon wafer with a period of 260 µm and a carrier lifetime of $\sim$ 1 µs.

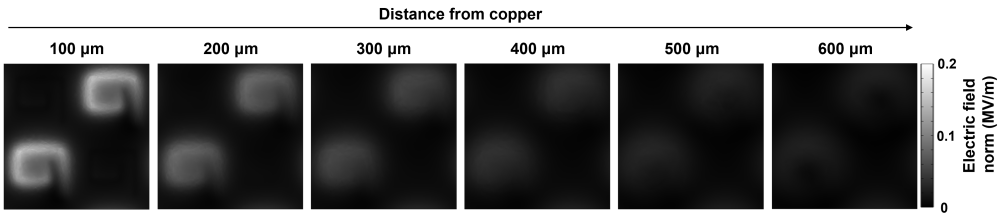


Fig. S5. Electric field norm at the lower frequency resonance (∼ 11.5 GHz without an air gap) in planes parallel to the copper layer at distances into the silicon layer as shown. Results of COMSOL simulation as described in Section 2.

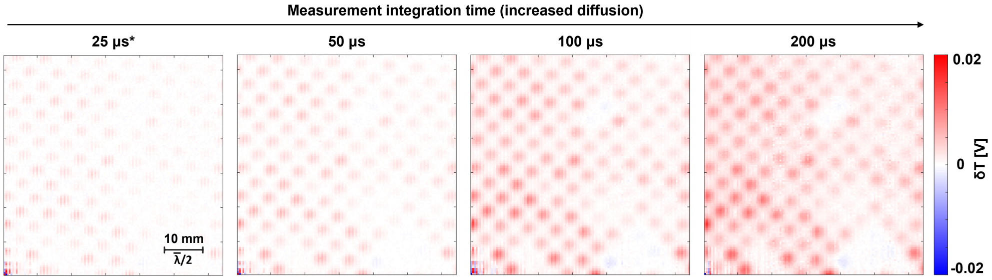


Fig. S6. The effect of pattern integration time, via changing both the DMD pattern hold time and measurement time, on the modulation depth and diffusion seen in microwave images. For the 25 μs exposure the DMD patterns were refreshed at the minimum 50 μs interval, with only the first half of the measured data used. The erroneous values in the lower left corner arise from stray DC signals in the values of **s**. $\bar{\lambda}$ denotes the wavelength at 15 GHz in free space.

thickness. This facilitates a significant improvement in resolution, as the effect of diffusion in the wafer is reduced due to its thickness and shorter charge carrier lifetime of approximately 1 μs. The calculated charge carrier distribution is shown in Fig. S4(c) and exhibits high contrast been the photoexcited and neighbouring pixels. The imaging resolution is therefore predicted to be limited by the projected pixel size of 130 μm in this case (50 μm region of Fig. 6 of the main text).

A 'soft' limit on the thickness of the wafer is imposed by the absorption of the visible pump light. A 6 μm thick wafer will absorb over 85% of an incident red pump light (after Fresnel losses)

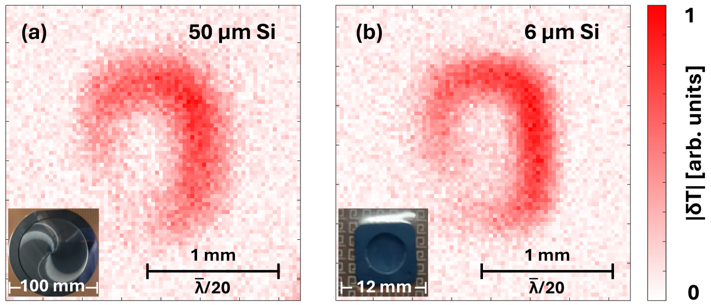


Fig. S7. Comparison between single-frequency imaging of a single meta-atom with a 50 μm and 6 μm thick silicon modulator, at 16.3 GHz and 18.5 GHz respectively. Images cropped from a larger 128 × 128 pixel image. Imaging times (number of averages, 200) and illumination intensity (1000 W/m$^2$) are the same for both. The silicon membranes are shown in the figure insets. In (b) a 600 μm dielectric overlayer with $\epsilon_r$ = 10.7 is used to reduce the native resonance frequency of the unloaded metasurface. $\bar{\lambda}$ denotes the wavelength at 15 GHz in free space.

following the parameters specified in Supplement 1 Section 3, and has been experimentally applied to near-field imaging [8]. To efficiently go thinner still, one could look to photo-excite above the direct band gap energy of silicon with wavelengths below ∼350 nm, where comparable absorption may be achieved in tens of nm.

We conclude our resolution discussion with a proof-of-principle imaging demonstration using an ultra-thin 6 μm thick photomodulator. By reconfiguring the projection optics for a smaller FOV, we increase the illumination intensity to 1000 W/m$^2$ and increase the number of images averaged to 200 to compensate for the reduced modulation signal. In Fig. S7 we compare a single frequency on-resonance image of a single meta-atom when using a 50 μm membrane and a 6 μm membrane. The 6 μm membrane is capable of a resolution of around 10 μm [8] but here is limited by the projection optics to ∼ 30 μm or $\bar{\lambda}/660$. Yet such deeply sub-wavelength resolution reveals no new information when compared with the 50 μm membrane ($\bar{\lambda}/165$). This highlights the importance of selecting the correct wafer and therefore resolution for a given task but also demonstrates that our technique has sufficient resolution capabilities to be applied throughout the millimeter wave and THz bands.

## 5. MODULATION DEPTH

The reduction in transmission predicted by the COMSOL modelling discussed in Section 2 is shown in Fig. S8, for a monochromatic photoexcitation intensity of 100 W/m$^2$ at 625 nm. This approximates the broadband experimental photoexcitation spectrum of the PE300BFM arc lamp, which is white with a heavy near IR supplement. The corresponding experimental modulation depth is measured with a VNA under flood illumination of ∼ 100 W/m$^2$ of the entire sample and is also shown in Fig. S8. The experimentally observed modulation is less than predicted, this may be due to a combination of factors. Firstly is the simplified treatment of the illumination spectrum in the modelling, which approximates a broad spectrum as monochromatic at 625 nm. Whilst we expect similar photon densities for the experimental illumination condition, this treatment neglects the wavelength dependent penetration depth of the components of the broad experimental lamp spectrum. In addition, we expect that the presence of the residual inhomogeneous broadening observed in Fig. 5 of the main text will reduce the observed modulation, as resonators shifted off the global resonance peak will be modulated less efficiently at the peak frequency.

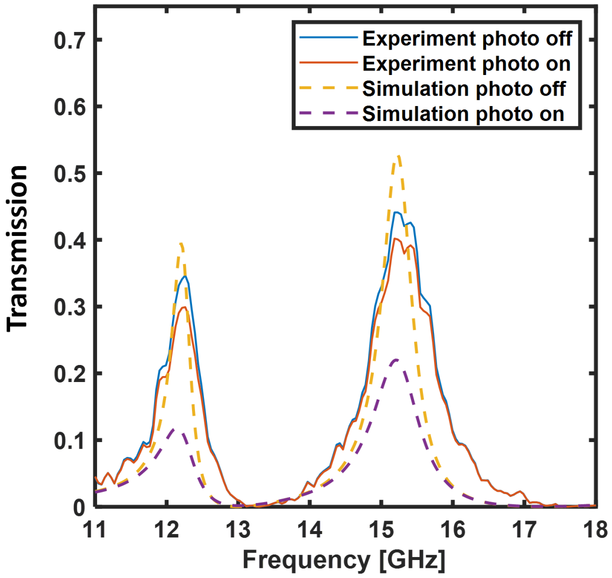


Fig. S8. Simulated effect of photomodulating the metasurface (with 1.7 μm air gap) with 100 W/m$^2$ of 625 nm monochromatic light, dashed lines. Experimental measurements for the sample imaged in Fig. 3 of the main text (after several weeks, showing some deterioration, see Section 6), taken with a VNA and under ∼ 100 W/m$^2$ of broad white - near IR illumination (PE300BFM spectrum).

## 6. REPEATED SAMPLE ASSEMBLIES AND FREQUENCY SAMPLING

One of the key advantages of the proposed system, vs mechanical near-field scanning probes, is its speed. To determine if resonators are active at the expected frequency requires only a single monochromatic image, which takes just 1.6 s even at very high 128 $\times$ 128 pixel resolution. We use this speed to confirm the nature of the defects in our layered metasurface. For completeness, we present the full (longer acquisition time) hyperspectral images in Fig. S9.

In Fig. S9(a) we image the same sample we imaged in the main text in Fig. 3 but with a reduced frequency spacing of 0.2 GHz and therefore acquisition time. As several weeks have passed since the sample was vacuum sealed, we notice some increased resonance shifting and dark regions, presumed due to air leakage or redistribution over time. In Fig. S9(b) we break the remaining vacuum seal, separate the metasurface layers and reseal the sample in a new vacuum bag without cleaning the surface. We see that the key centers for the defects remain in place (the FOV alignment of the images is not identical), but that the exact shape and degree of spectral broadening is reduced. This clearly rules-out fixed geometric or material factors such as the spiral lengths or substrate permittivity (alone) being the cause. We understand the defect centers to be solid contaminants and the surrounding variable broadening to be due to trapped air.

In Fig. S9(c) we clean the mating surfaces of the metasurface and the wafer with fairly aggressive mechanical action and isopropyl alcohol as a solvent. We observe that the majority of the defects are removed, confirming them to be interfacial in nature.

In Fig. S9(c) we also see that our imaging method is also able to detect the spatial variations in signal that arise from the presence of experimental artefacts such as standing waves, manifesting as broad vertical stripes in the images (which are typically observed as spurious rapid oscillations in conventional VNA spectra). This vertical banding may also be observed in the monochromatic images of figures S13 and S14 (in particular Fig. S14 14.7-14.9 GHz). At nearby imaging frequencies ($\sim$100 MHz, comparable to the period of standing wave oscillation), this can give rise to the appearance of complementary regions similar to those in Fig. 2(b) and (c) of the main text but these are distinct in spatial character and frequency spacing from the defects we discuss in this work. In our hyperspectral images these artefacts will be effectively averaged out as long as spectral sampling of the imaging is sufficient as shown in Fig. S9(d).

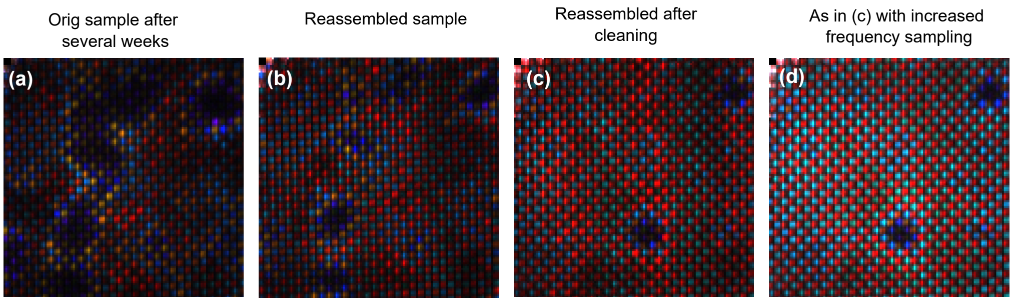


Fig. S9. Spatial variation in interfacial defects observed for samples (re)assembled under different conditions, with the same colour to frequency mapping as in Fig. 3 of the main text. (a) The sample from the main text Fig. 3, re-imaged after several weeks with some apparent degradation and with reduced frequency sampling (0.2 GHz increments). (b), as in (a) but the sample was disassembled and resealed. (c) as in (b) but the mating surfaces were first thoroughly cleaned with isopropanol. (d) as in (c), but with (sufficient) frequency sampling of 0.1 GHz.

## 7. MINIMALLY PERTURBATIVE IMAGING OF AN ARBITRARY METASURFACE

For imaging metasurfaces specifically, perturbation of the local fields is generally a far more important effect than achieving highly subwavelength resolution, as the modulator thickness and location also influence the response of the metasurface itself. Perturbation is a well known problem in metasurface imaging: for example, tips used for efficient near field scattering often perturb the very fields they are trying to characterise. As such, it is an aspiration of all near field imaging approaches to be as minimally perturbative as possible.

One approach towards minimal perturbation is to reduce the modulator thickness. This is demonstrated through simulations of a simple dipolar metasurface that is loaded on one side by

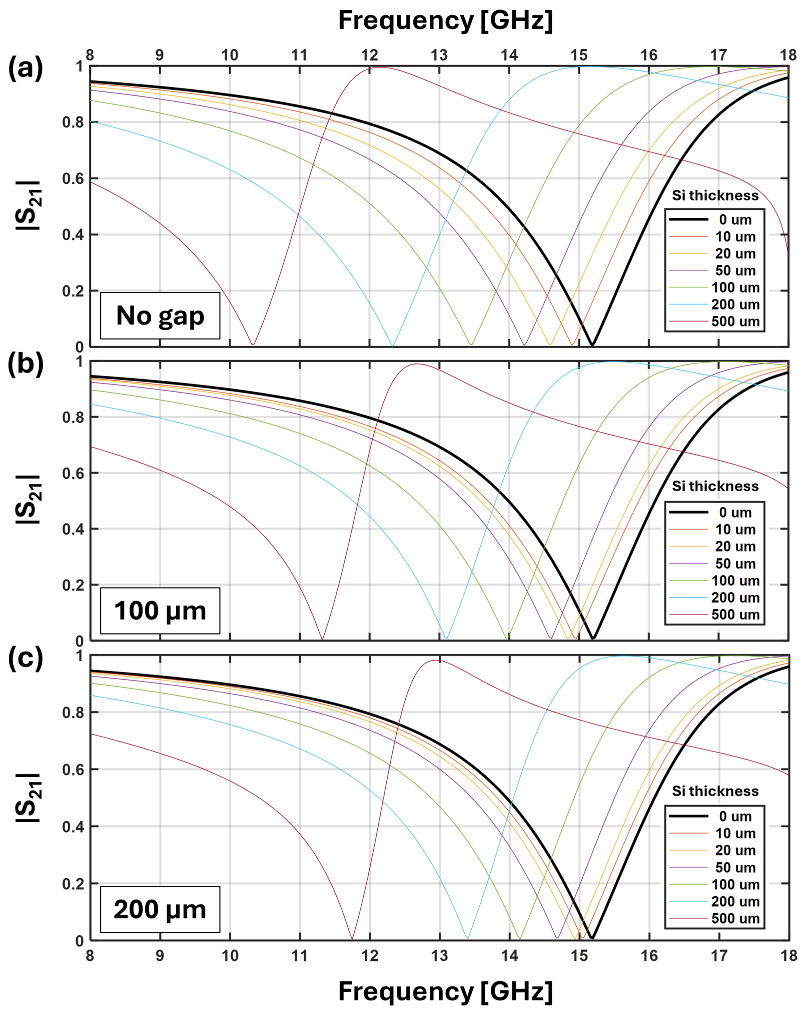


Fig. S10. (a) Results of simulations of the dipolar metasurface geometry shown in Fig. S11, with varying thicknesses of silicon photomodulator included as indicated. Square root of the normal incidence transmission as a function of frequency. (b) and (c), as in (a) but including an air gap between the metasurface and modulator of 100 and 200 μm respectively.

an extrinsic variable thickness silicon layer. In Fig. S10(a), we see that reducing the added silicon photomodulator thickness is an effective way of converging the perturbed resonance frequency to the unloaded case. By reducing the total overlap of a meta-atom's near fields with the modulator layer, the resonance frequency remains much closer to the unloaded metasurface. The model file for this simulation, performed using Ansys® Electronics Desktop HFSS 2021 R1, is included in the data set accompanying this work, from https://doi.org/10.5281/zenodo.15208969 and the model geometry can be seen in Fig. S11.

This effect is demonstrated experimentally using a large area silicon membrane In Fig. 6 of the main text. In general, the thinner the membrane, the smaller the impact on the intrinsic functionality of the metasurface under investigation. As discussed in Supplement 1 Section 4, a 'soft' limit on the thickness of the wafer is imposed by the absorption of the visible pump light. A 6 μm thick wafer will absorb over 85% of an incident red pump light (after reflection losses) following the parameters specified in Supplement 1 Section 3, and has been experimentally applied to near-field imaging [8]. To efficiently go thinner still, one could look to photo-excite above the direct band gap energy of silicon with wavelengths below ∼350 nm, where comparable absorption may be achieved in tens of nm.

A second approach towards minimal perturbation is to place the modulator in the tails of the

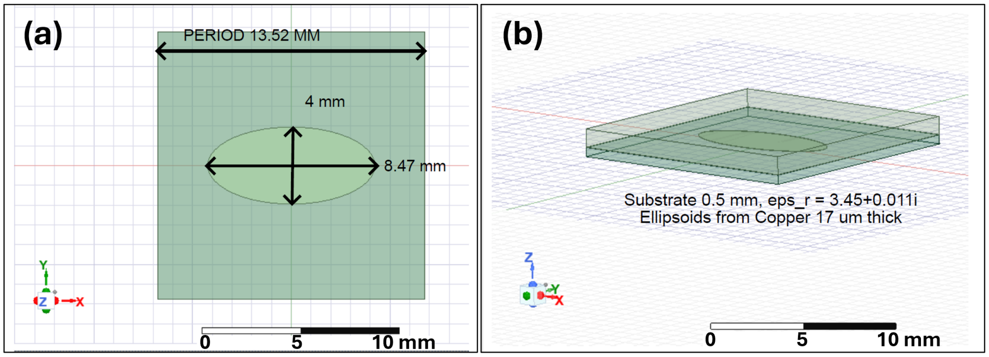


Fig. S11. Ansys HFSS periodic model geometry. The copper ellipse sits atop a 0.5 mm dielectric substrate and then an additional silicon layer sits on top of the ellipse, with thickness and gap size as indicated in the results of Fig. S10. The permittivity of silicon used is 11.7+0.003i. Images used courtesy of ANSYS, Inc.

meta-atom evanescent fields - this is the approach typically used in tip scattering imaging to reduce perturbation effects. Increasing the distance between the modulator and the meta-atoms is a simple but effective way to reduce the spectral shift introduced by its presence. Furthermore an offset may be necessary in multilayer metasurfaces or due to the presence of encapsulant layers. This effect is shown through simulation in Fig. S10(a-c), as an increasing air gap is added

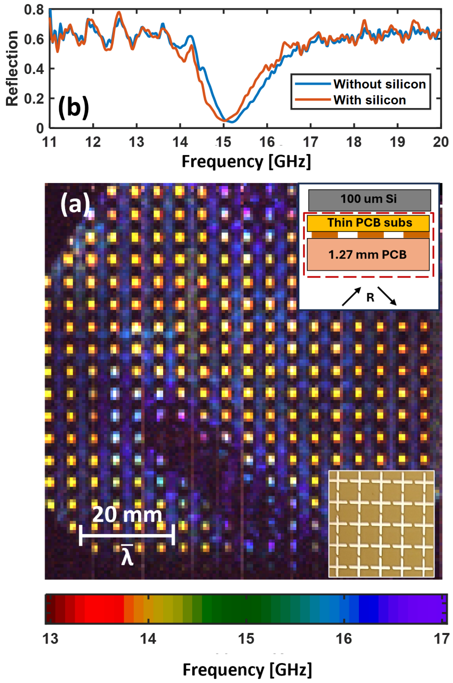


Fig. S12. (a) Hyperspectral image (TE, 35 ° reflection) of a simple array of crossed dipole meta-atoms containing no intrinsic silicon (copper rectangles as shown in lower inset of (a)). The 100 μm thick passivated silicon modulator is introduced to the layered metasurface (red dashed box) as shown in the upper inset of (a) and then the structure is vacuum sealed for improved uniformity. In (b), VNA reflection spectra before and after the introduction of the silicon reveal that the spectral response of the metasurface is almost entirely unperturbed. The 'thin PCB' substrate in the figure inset is 0.28 mm thick. The monochromatic images for the full data set can be seen in Fig. S18. $\bar{\lambda}$ denotes the wavelength at 15 GHz in free space.

between the original metasurface and the extrinsic silicon modulator. However, this approach has important trade-offs in terms of both modulation efficiency and imaging resolution.

Fortunately, a reduced modulation efficiency can be compensated for by increasing the lifetime of the charge carriers in the silicon (or the illumination intensity). In Fig. S12 we demonstrate minimally perturbative imaging of a new, non-photoactive metasurface by means of introducing a 100 μm thick double-sided passivated silicon wafer with a carrier lifetime of 1.3 ms. The surfaces of the wafer have been treated with $Al_2O_3$ to significantly reduce surface recombination [9]. The metasurface design is shown in the figure insets consisting of dipolar copper crosses with 4-fold symmetry. The unperturbed metasurface (dashed red box inset top left) supports a resonance at $\simeq$15.2 GHz, shown in the VNA reflection spectrum in (b). When loaded with the thin silicon 280 um away from the layer of metallic meta-atoms (on top of a thin PCB layer intrinsic to the metasurface), the shift in resonance frequency is as small as 200 MHz, as shown in (b). Crucially, the hyperspectral image (a) still shows excellent contrast, throughput and resolution (as diffractive effects are small over this offset distance). One can clearly see the individual dipolar fields of the vertical metallic bars as well as defective regions caused by over etching of the copper during fabrication.

Finally,it should be noted that the perturbative effects arising from the silicon modulator (a single, homogeneous layer) are significantly easier to calculate accurately (and therefore mitigate) than for common near field imaging approaches e.g. employing tip scattering.

## 8. IMAGING RESULTS: SINGLE-FREQUENCY IMAGES

The hyperspectral images presented in the main text are created from data cubes that extend in the spatial $x$ and $y$ directions and in the frequency domain (see the inset of Fig. 2(a) of the main text), obtained by single-pixel imaging at multiple frequencies in turn. A mapping between microwave frequency and visible colours is generated and follows the colourbars accompanying Figures 3-6 of the main text. The final image pixel's colour and brightness is determined by summing contributions from each of the monochromatic microwave images (with their own associated colour) at that pixel location. For clarity and to increase the accessibility of this large, information rich data set, the individual monochromatic microwave image sets for the main results of this paper are shown in the following figures.

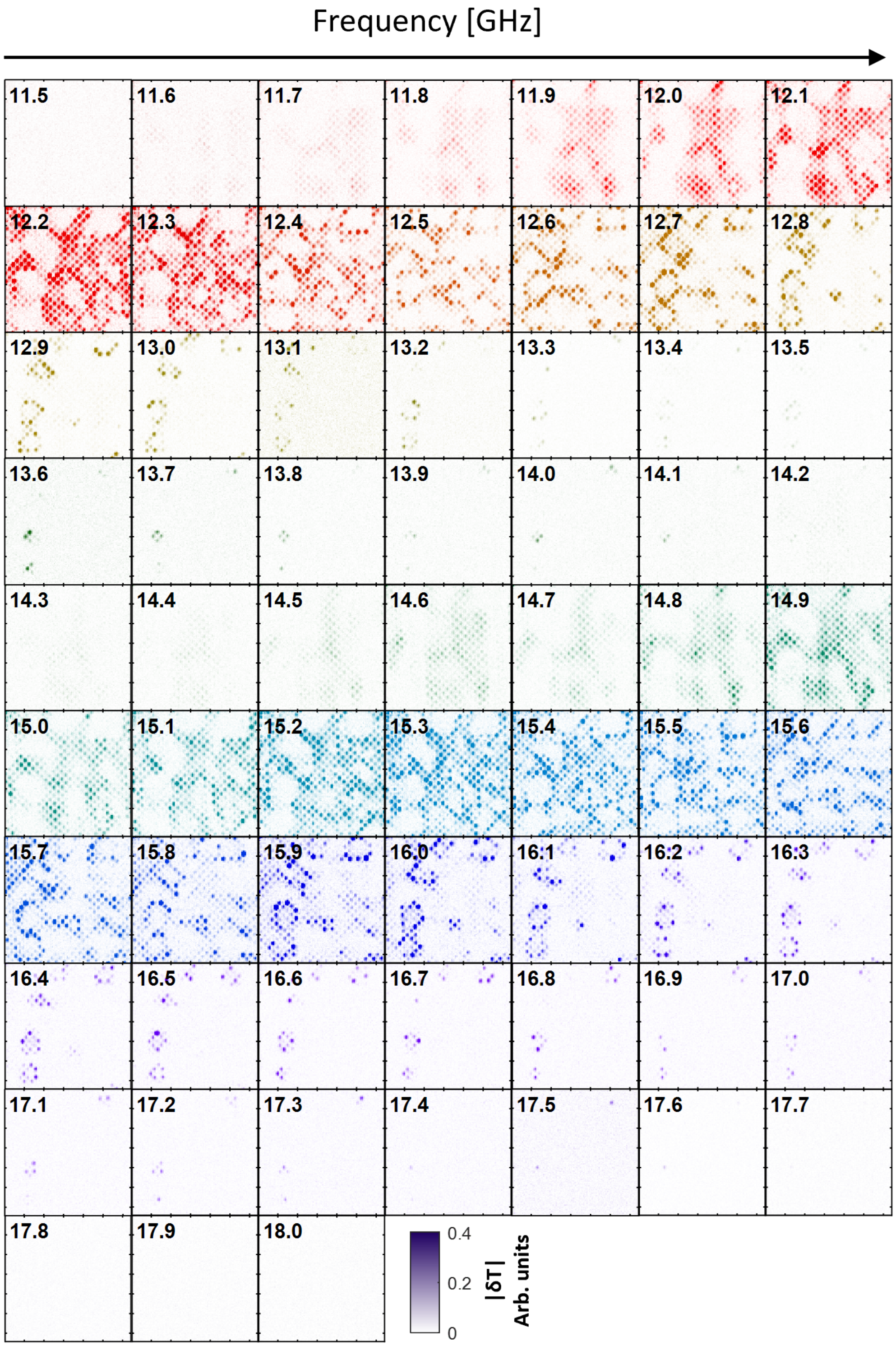


Fig. S13. Single frequency images, as labelled, for the dataset forming the hyperspectral image of Fig. 3 of the main text. Frequencies containing no resonators have been omitted from either end of the dataset to save space. The colourbar applies to the colour saturation of each monochromatic image.

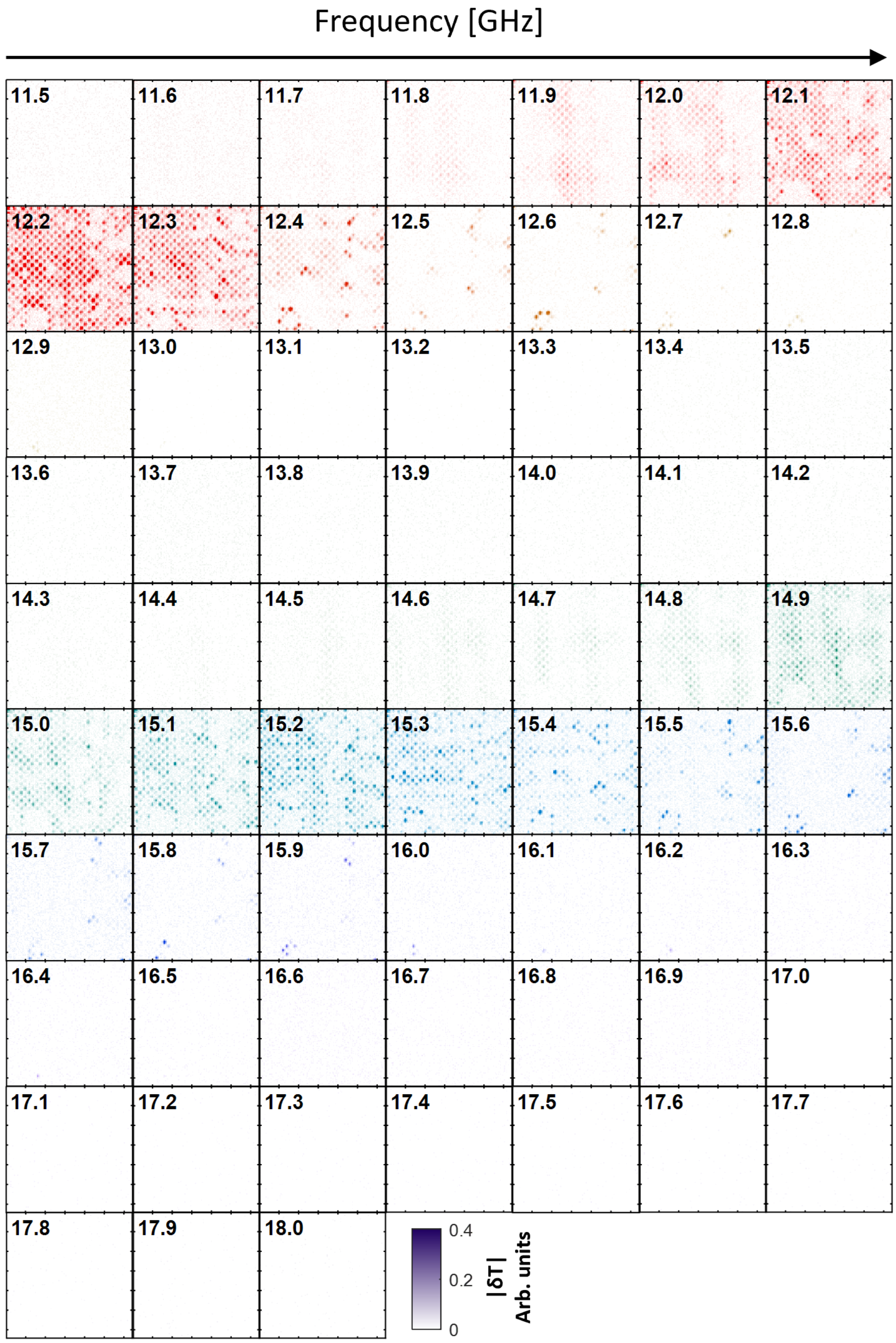


Fig. S14. Single frequency images, as labelled, for the dataset forming the hyperspectral image of Fig. 5 (reduced inhomogeneous broadening) of the main text. Frequencies containing no resonators have been omitted from either end of the dataset to save space. The colourbar applies to the colour saturation of each monochromatic image.

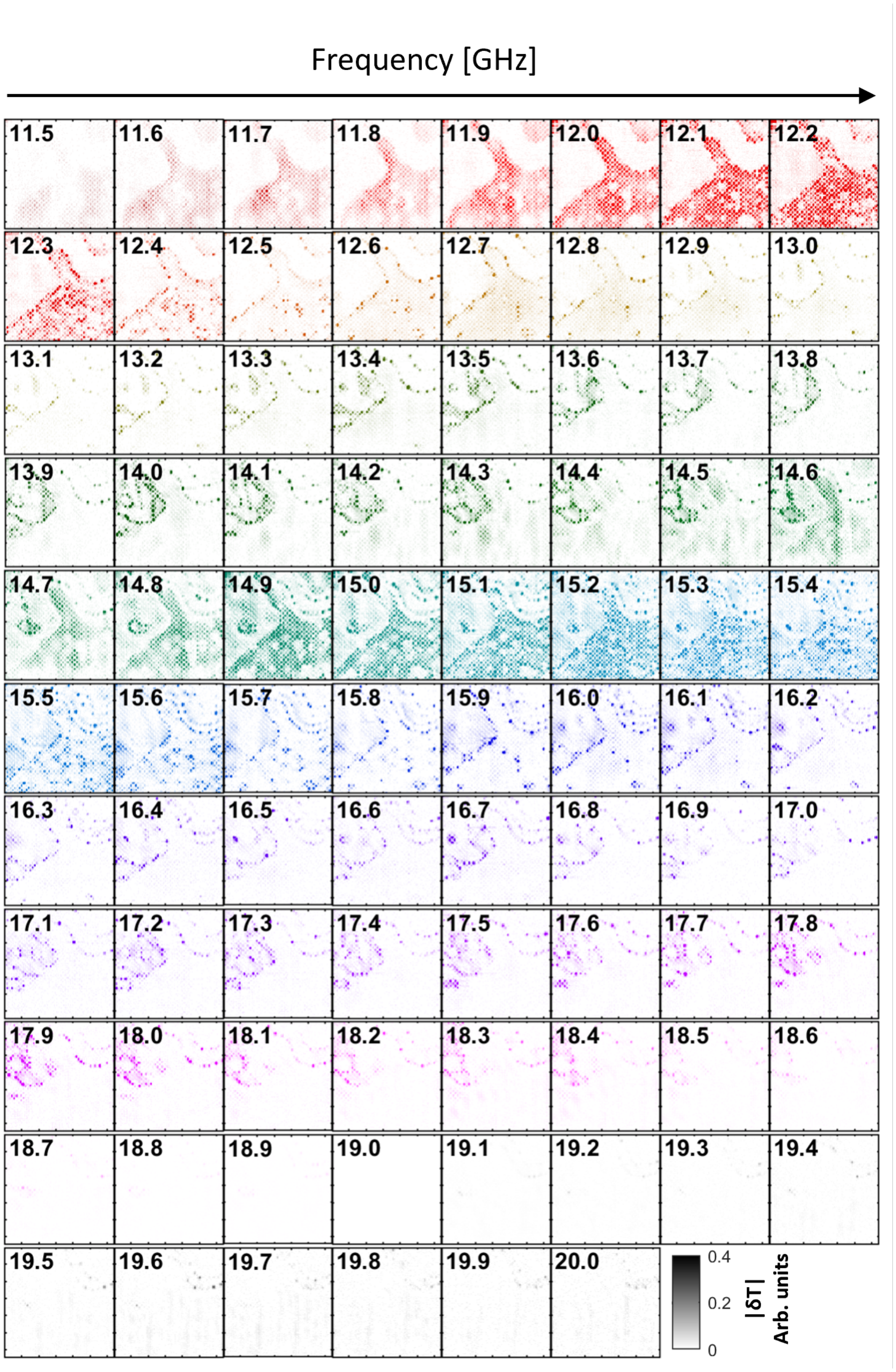


Fig. S15. Single frequency images, as labelled, for the dataset forming the hyperspectral image of Fig. 4(a) (intentional defects) of the main text. Frequencies containing no resonators have been omitted from either end of the dataset to save space. The colourbar applies to the colour saturation of each monochromatic image.

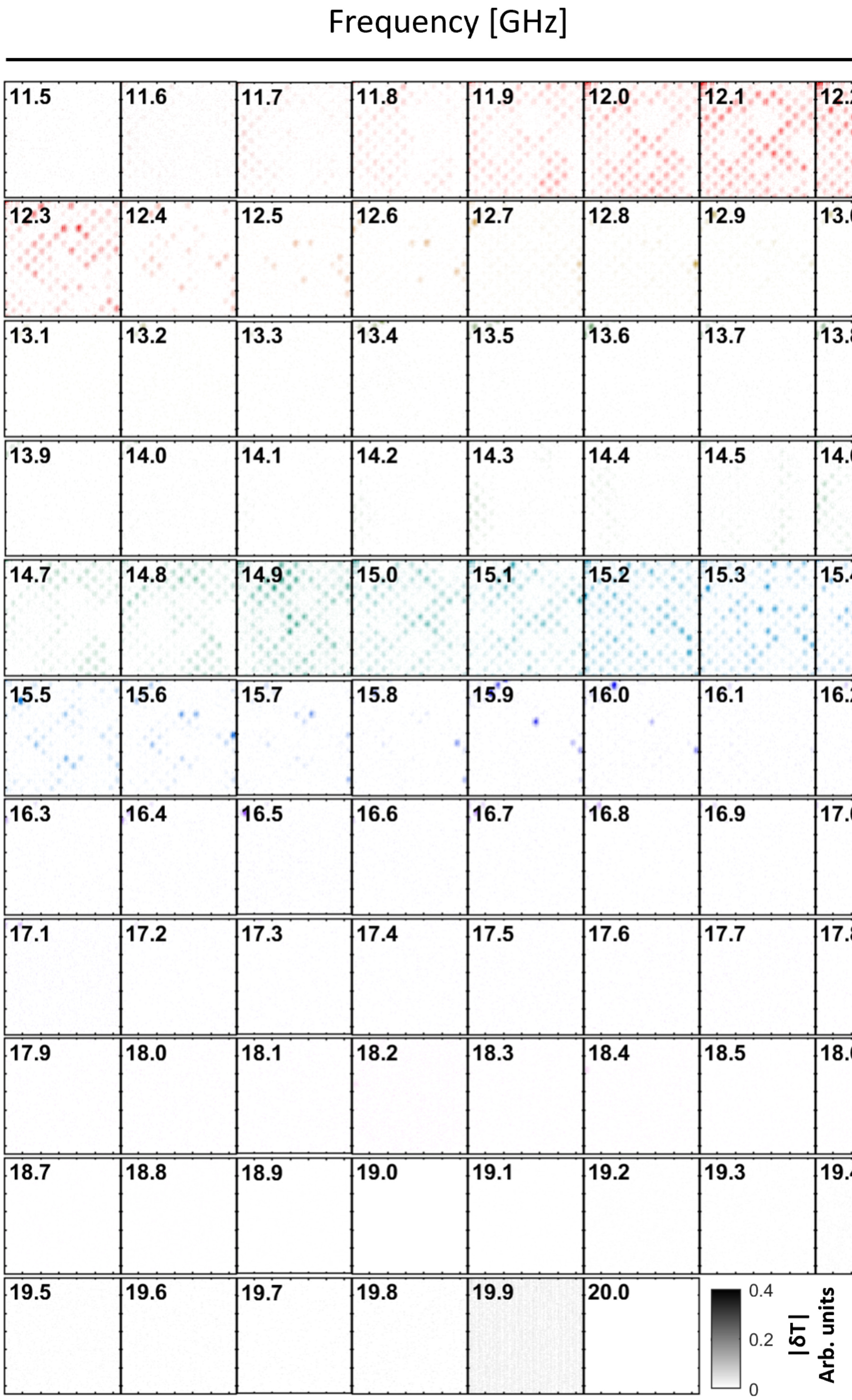


Fig. S16. Single frequency images, as labelled, for the dataset forming the hyperspectral image of Fig. 4(b) (small FOV of intentional defects) of the main text. Frequencies containing no resonators have been omitted from either end of the dataset to save space. The colourbar applies to the colour saturation of each monochromatic image.

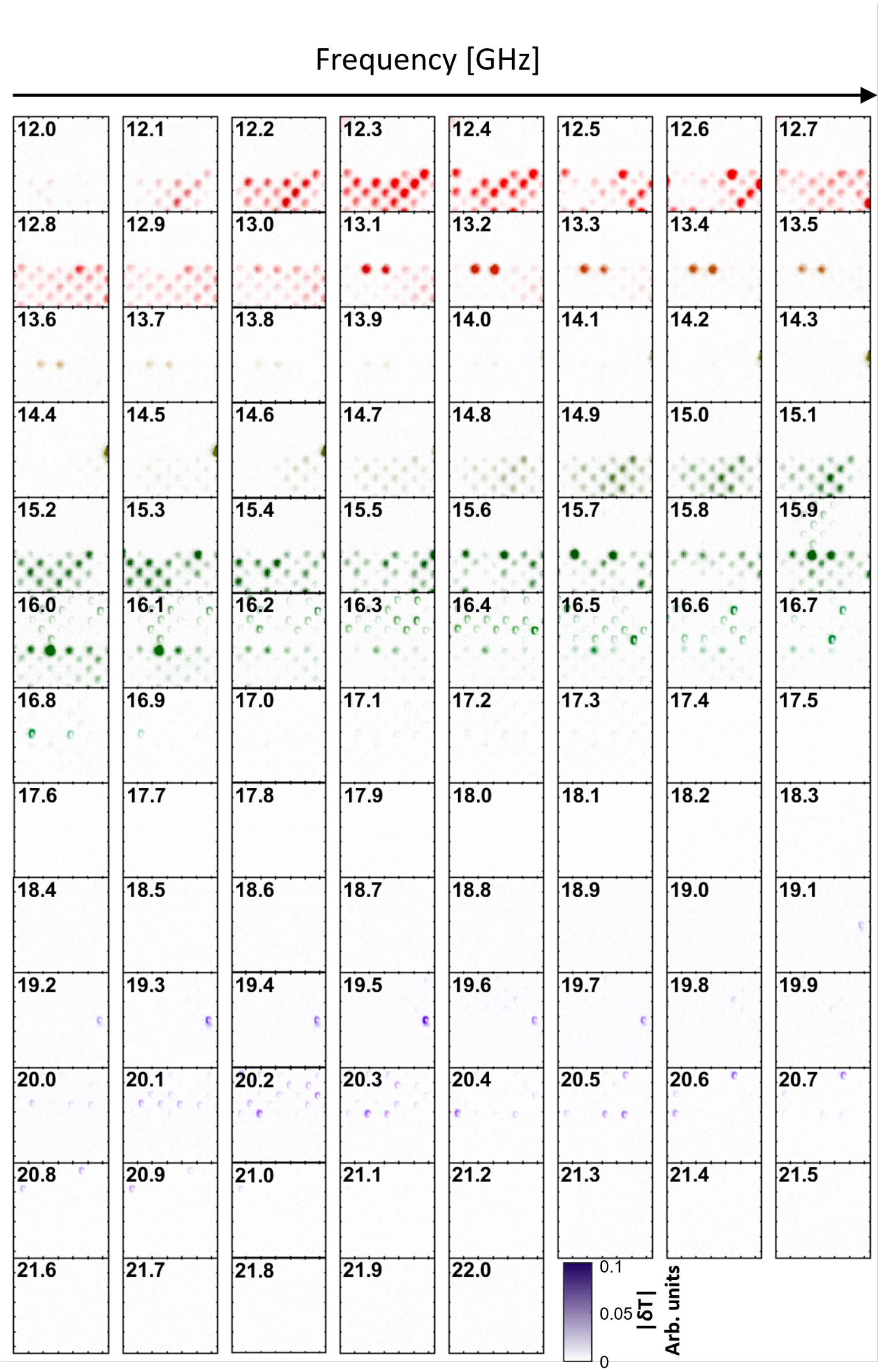


Fig. S17. Single frequency images, as labelled, for the dataset forming the hyperspectral image of Fig. 6 of the main text. The colourbar applies to the colour saturation of each monochromatic image.

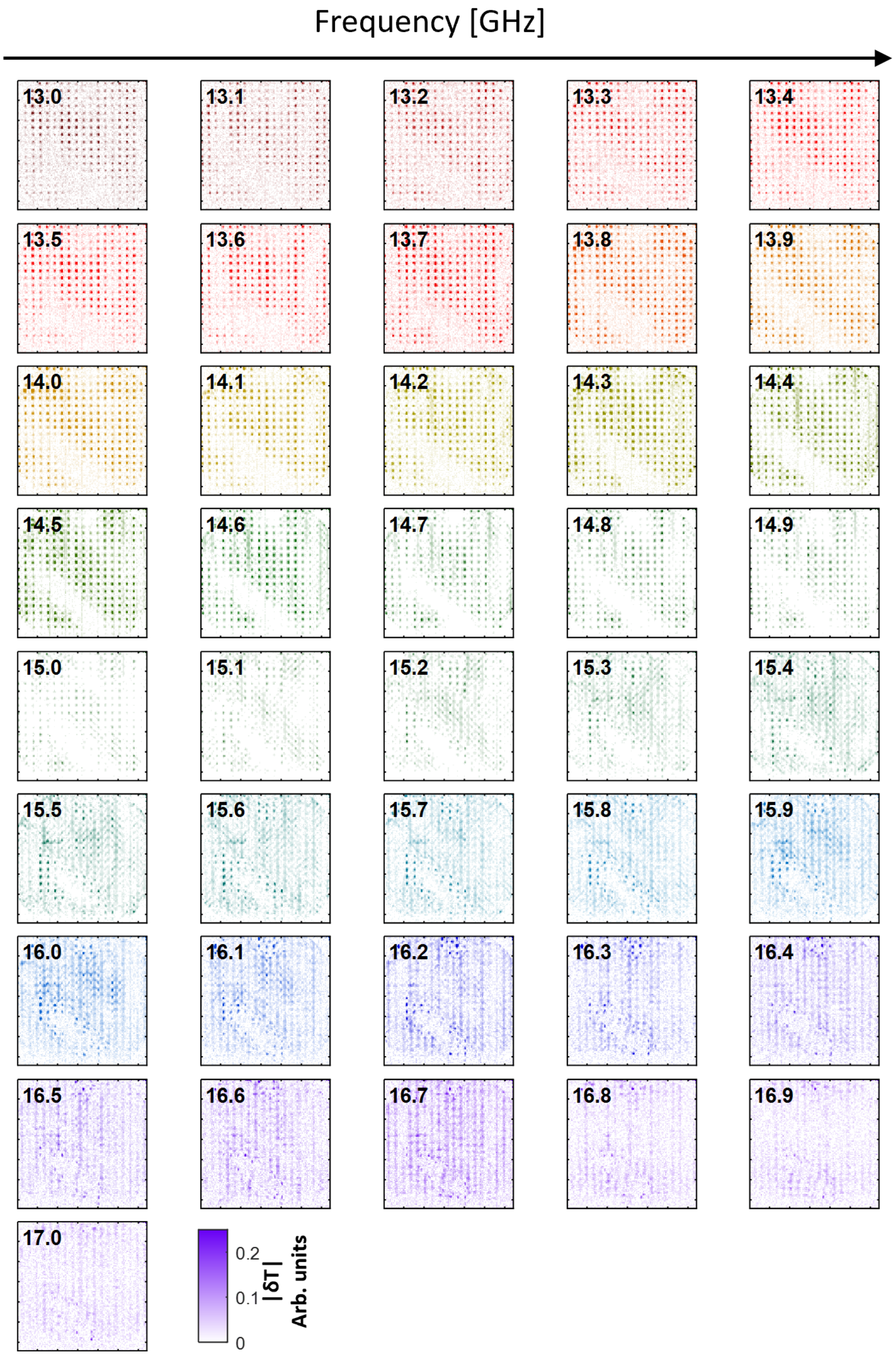


Fig. S18. Single frequency images, as labelled, for the dataset forming the hyperspectral image of Fig. S12. The colourbar applies to the colour saturation of each monochromatic image.

## REFERENCES


1. M. J. Padgett and R. W. Boyd, "An introduction to ghost imaging: Quantum and classical," Philos. Transactions Royal Soc. A: Math. Phys. Eng. Sci. **375** (2017).
2. G. M. Gibson, S. D. Johnson, and M. J. Padgett, "Single-pixel imaging 12 years on: a review," Opt. Express **28**, 28190 (2020).
3. H. Penketh, W. L. Barnes, and J. Bertolotti, "Implicit image processing with ghost imaging," Opt. Express **30**, 7035 (2022).
4. N. J. Sloane, "Multiplexing methods in spectroscopy," Math. Mag. **52**, 71–80 (1979).
5. M. Harwit, Hadamard transform optics (Elsevier, 2012).
6. I. R. Hooper, N. E. Grant, L. E. Barr, et al., "High efficiency photomodulators for millimeter wave and THz radiation," Sci. Reports **9** (2019).
7. B. Huang and Q. Jia, "Accurate modeling of conductor rough surfaces in waveguide devices," Electronics **8** (2019).
8. R. I. Stantchev, D. B. Phillips, P. Hobson, et al., "Compressed sensing with near-field THz radiation," Optica **4**, 989 (2017).
9. I. R. Hooper, E. Khorani, X. Romain, et al., "Engineering the carrier lifetime and switching speed in si-based mm-wave photomodulators," J. Appl. Phys. **132**, 233102 (2022).
